# Demonstrative Midwifery

**Published:** 1850-06

**Authors:** 


*Demonstrative Midwifery.*—The following note has been handed to us with a request that it be inserted in this Journal. We comply with the request with pleasure, reserving, for a subsequent No., a review of the correspondence contained in the March No. of the last volume of this Journal, and a discussion of the merits of the subject in general. In connection with a future article on the subject, we shall present our readers with the opinions expressed by contemporaneous medical journals, respecting the "innovation" of connecting demonstrative with didactic instruction, in the department of Obstetrics ; and respecting, also, the letter contained in our March No., rebuking the practice pursued at the Medical Department of the University of Buffalo. In the letter accompanying the following note, it is stated that the letter expresses the *unanimous* opinion of the profession, so far as Racine is concerned.

Racine, Wisconsin, May 10th, 1850.

Prof. J. P. White—

*Dear Sir :* The undersigned, practising Physicians in this city, having perused the correspondence relative to the introduction of demonstrative Midwifery in the University of Buffalo, take pleasure in expressing to you their unqualified approbation of the course pursued in the department of Obstetrics.

Allow us, Sir, to hope and expect, that the work so nobly begun will be steadily prosecuted, until the medical student shall have ample opportunity to qualify himself in *all* the duties of his profession.

| | |
|---|---|
| WM. WATKINS, | P. LAURENCE PAGE, |
| AUG. H. HANCHETT, | SAM'L W. WILSON. |
| B. B. CAREY, | E. JAMIESON, |
| JOSEPH B. TALCOTT, | EDWARD EVERITT, |
| W. WADSWORTH, | S. H. GRAVES. |

